# A Simple Test to Detect Deltoid Function

**DOI:** 10.1055/s-0045-1810111

**Published:** 2025-08-06

**Authors:** Anil Bhatia, Alex Franco de Carvalho, Ruy Dantas Silveira Gois Neto

**Affiliations:** 1 Department of Brachial Plexus Surgery, Deenanath Mangeshkar Hospital and Research Center, Pune, Maharashtra, India; 2 Northeast Center of Plastic and Reconstructive Microsurgery, Brazil; 3 Department of Hand and Microvascular Surgery, Federal University of Sergipe, Sergipe, Brazil

Assessment of deltoid reinnervation following brachial plexus injury remains a clinical challenge. Traditional tests, such as the Bertelli abduction-in-internal-rotation test, may yield false-negative results due to compensatory activation of adjacent muscles. Concomitant rotator cuff dysfunction may interfere with testing. Electromyography (EMG) has not proved to be efficient to document early signs of reinnervation of the posterior fibers of the deltoid.

We describe a straightforward clinical test that allows the examiner to isolate and confirm voluntary contraction of the posterior deltoid. This test has been applied consistently over years of clinical follow-up and is applicable in cases of nerve grafting, nerve transfers, or spontaneous reinnervation of the axillary nerve.

## Materials and Methods



**Video 1** Video of a normal individual showing isolated contraction of the deltoid.


**Video 2** Contraction of posterior fibers of the deltoid indicating early re-innervation.


The patient is examined while upright (sitting or standing). The arm rests on a flat platform at 90 degrees abduction in the coronal plane. He/she is asked to lift the elbow off the platform. The examiner, standing behind the patient, then observes for contraction of the deltoid.

A positive test is defined by visible contraction of the posterior deltoid fibers ( Videos 1 and 2 ). Notably, no resistance is applied. The test is applicable irrespective of the reconstructive technique used (nerve grafts, transfers, or conservative observation).

## Discussion

**Fig. 1:**
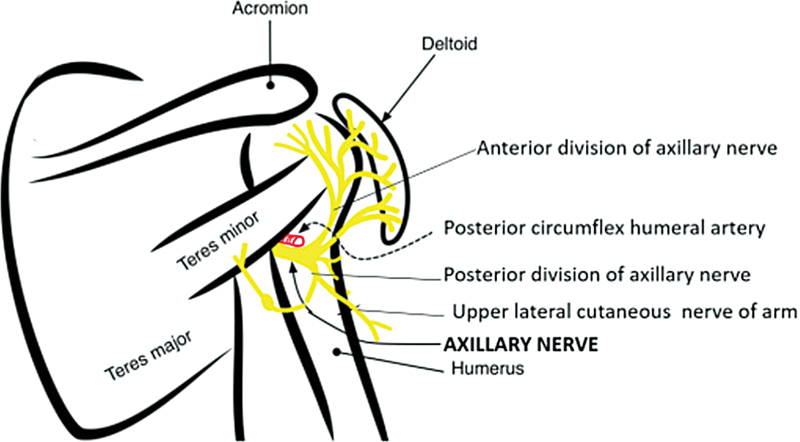
The axillary nerve traverses the quadrangular space and enters the deltoid muscle from the back innervating the posterior fibers first.

Evaluation of the function of the deltoid is challenging. Attempted abduction inevitably involves use of the rotator cuff. Bertelli's test has proved useful in detecting isolated deltoid deficit in the presence of an intact rotator cuff. However, there are clinical situations where the test has not proved useful in guiding clinical strategy. For instance, infraclavicular brachial plexus injuries following trivial trauma often present with axillary nerve injuries. In such cases, the impact is at a low velocity and spontaneous recovery is anticipated. However, abduction in internal rotation cannot be administered because of the rotator cuff weakness. The axillary nerve enters the deltoid from the posterior margin and those fibers will contract first ( Fig. 1 ). This test has proved uniformly successful in detecting the contraction of the deltoid so that further recovery can be expected and unnecessary nerve transfers can be avoided. Similarly, detection of this reinnervation on an EMG will also involve specific activation of the deltoid when the needle is inserted. So this report can also serve as a guideline for more accurate electrodiagnostic evaluation in such patients. This test addresses key limitations in the clinical evaluation of shoulder abduction following brachial plexus injury. By requiring only conscious activation of the posterior deltoid at 90-degree abduction, it isolates the muscle effectively without requiring full limb elevation or rotator cuff support. Thus, reinnervation of the deltoid following a nerve transfer or grafting procedure can be demonstrated. If a mirror is employed, it serves as a biofeedback device to encourage strengthening of the deltoid.

Limitations include the absence of interobserver validation and formal quantification of sensitivity/specificity.

## Conclusion

This maneuver provides a fast, reliable, and specific method to confirm isolated posterior deltoid reinnervation across a wide range of brachial plexus reconstructive strategies. It should be considered a valuable addition to both clinical evaluation and electromyographic assessment of deltoid function.

## References

[1] Bertelli J A Ghizoni M F Abduction in internal rotation: a test for the diagnosis of axillary nerve palsy J Hand Surg Am 2011 36 12 2017 2023 22051233 10.1016/j.jhsa.2011.09.011

